# Clinical outcomes of aortic root repair using pericardial autograft for acute type a aortic dissection

**DOI:** 10.1186/s13019-024-02909-2

**Published:** 2024-06-26

**Authors:** Chuang Liu, Yao Wang, Peigang Ouyang, Yangxue Sun, Lingchen Huang, Xiaogang Sun, Xiangyang Qian, Cuntao Yu, Hongwei Guo

**Affiliations:** 1 Department of Cardiovascular Surgery, Fuwai Hospital, Chinese Academy of Medical Sciences and Peking Union Medical College, No 167, Beilishi Road, Beijing, 100037 China; 2 Department of Cardiovascular Surgery, Fuwai Hospital Chinese Academy of Medical Sciences, Shenzhen, China; 3 Department of Cardiac Surgery, Yunnan Fuwai Cardiovascular Hospital, Kunming Medical University, Kunming, China

**Keywords:** Acute type A aortic dissection, Aortic root repair, Pericardial autograft, Aortic sinus

## Abstract

**Background:**

For acute type A aortic dissection involving the aortic root with root diameter no more than 45 mm, there are various aortic root repair techniques. In this study, a novel surgical technique using a pericardial autograft for aortic root repair was introduced. We described its surgical steps in detail and compare its clinical outcomes with direct suture technique.

**Methods:**

Between July 2017 and August 2022, 95 patients with acute type A aortic dissection who underwent aortic root repair were enrolled, including aortic root repair using pericardial autograft (group A, *n* = 49) or direct suture (group B, *n* = 46). The patient’s clinical data were retrospectively analyzed, and a 5-year follow-up was conducted.

**Results:**

The 30-day mortality, re-exploration for bleeding, postoperative new-onset renal failure requiring continuous renal replacement therapy, stroke, and paraplegia occurred in 3%, 4%, 11%, 5%, and 2% of the overall patients, respectively. There was no significant difference in the 30-day mortality and complication rate between the two groups. The 30-day mortality and re-exploration for bleeding marked the primary endpoint events. Logistic regression analysis indicated that there was a significant correlation between the primary endpoint events and surgical technique (odds ratio, 0.002; 95% confidence interval, 0-0.159; *P* = 0.026). The aortic valve insufficiency of the two groups were significantly improved after operation (group A, *P* < 0.001; group B, *P* < 0.001). During follow-up, there was no significant difference in short-term survival between the two groups after surgery (log-rank *P* = 0.75), and all patients were free from reoperation for aortic disease.

**Conclusions:**

Patients who underwent aortic root repair using pericardial autograft tended to have reduced 30-day mortality and a lower risk of re-exploration for bleeding. Using pericardial autograft for aortic root repair is a safe and useful approach for patients with acute type A aortic dissection involving the aortic root.

**Supplementary Information:**

The online version contains supplementary material available at 10.1186/s13019-024-02909-2.

## Introduction

Acute Stanford type A aortic dissection (ATAAD) is characterized by an acute onset, severe conditions, and high mortality rates [1]. Emergency surgery is essential once the diagnosis is made. However, treating aortic dissection becomes challenging when the aortic root is affected, leading to the avulsion of aortic valve commissures and involvement of coronary ostia [2]. In ATAAD patients with aortic root diameter no more than 45 mm, aortic root repair with resuspension of the aortic valve and obliteration of the false lumen is appropriate [1]. Various aortic root repair techniques are used, including the sandwich and modified sandwich techniques [3], the adventitial inversion technique [4], the neo-media technique [5], and the direct suture technique [6]. Typically, it is inevitable to preserve a portion of the dissected intima and utilize artificial materials in these aortic root repair techniques, which can elevate the risk of proximal anastomosis bleeding, cause stiffness or distortion of the aortic root, and negatively impact its durability and physiological function [4, 6]. 

To address these challenges, we have been performing aortic root repair using pericardial autograft in recent years. This approach aims to overcome the aforementioned issues by completely removing the dissection tissue and preserving the physiological function of the aortic root [7]. However, the research regarding the clinical outcomes of this novel surgical technique is insufficient. Hence, we aim to investigate our initial experience and evaluated the short-term clinical outcomes of this new technique in comparison to direct suture technique.

## Methods

### Ethics

This study conformed to the principles outlined in the Declaration of Helsinki. This study was approved by the ethics committees of our hospital (No. 2021 − 1490, approved on Dec 6th, 2021), and written informed consent was waived due to the retrospective nature of the study. This study was a single-center, retrospective observational cohort study.

### Patients

A total of 95 ATAAD patients with aortic root involvement, who underwent aortic root repair at our institution from July 2017 to August 2022, were included in the study. Patients with ATAAD involving the aortic root frequently have the avulsion of aortic valve commissures and involvement of coronary ostia [Figure S1]. The aortic arch of all patients was involved to varying degrees. Patients who underwent aortic root repair using pericardial autograft were assigned to group A, while those who received the direct suture technique were assigned to group B. The inclusion criteria were as follows: (1) patients aged 18–80 years; and (2) ATAAD patients with aortic root involvement. Exclusion criteria included: (1) patients with severe coagulation abnormalities, (2) enlarged aortic root (aortic root diameter > 45 mm), and (3) patients diagnosed with Marfan syndrome or other hereditary thoracic aortic disorders. The surgical technique for ATAAD patients is on the discretion of surgeons. In this study, direct suture was mainly performed in 2019 and before. Since 2020, we have proposed and improved aortic root repair using pericardial autograft. Therefore, since 2020, all patients treated by our team have undergone our new surgical technique, provided that their conditions permit.

### Surgical technique

For patients in group A, we elaborate on the method of aortic root repair using pericardial autograft in detail.

**Fig. 1:**
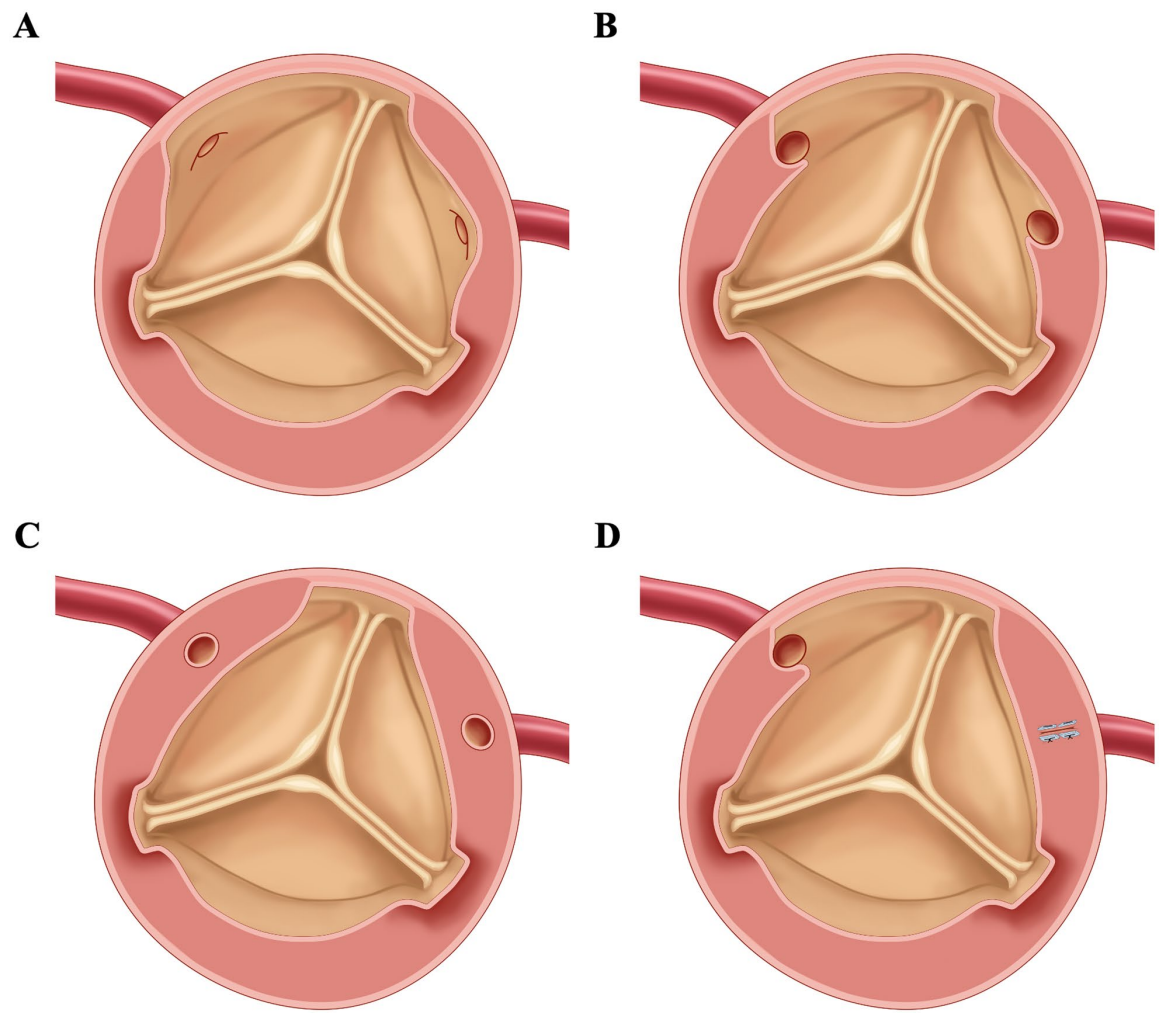
(**A**) to (**C**) Excision of dissected intima where coronary ostia are not (**A**), partially (**B**) or totally (**C**) involved, and preservation of intimal flap near the avulsed commissures. (**D**) Closure of the impaired coronary arterial orifice where the coronary artery intima is transected or torn beyond the coronary ostia

Removal of the dissected intima: the dissected intima was completely excised until reaching the normal aortic wall. A fresh pericardial autograft without glutaraldehyde treated was used to reconstruct the aortic root, while preserving the intimal flap located 5 mm away from the avulsed commissures [Fig. 1A]. In cases where the coronary ostia were partially or totally involved, the dissected intima flap adjacent to the coronary artery was also removed until reaching the normal coronary ostia [Fig. 1B and C]. Moreover, if the coronary artery intima was transected or torn beyond the coronary ostia, repairing the coronary ostia was not feasible. In such cases, the impaired coronary arterial orifice was closed, and a coronary artery bypass graft (CABG) was performed [Fig. 1D].

**Fig. 2:**
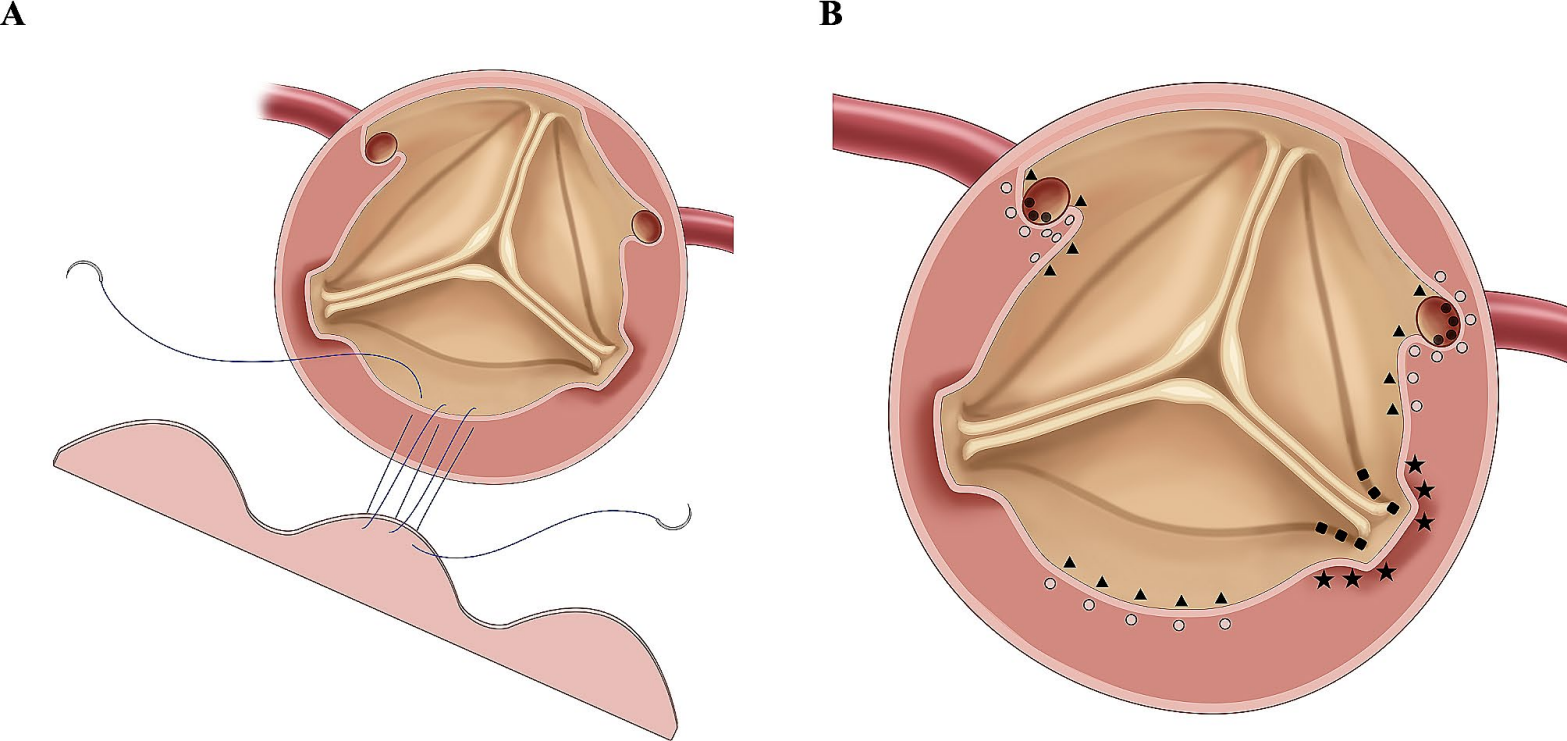
(**A**) The trimmed aortic wall and a tailored pericardial patch are sutured continuously, starting from the lowest point of the noncoronary sinus. (**B**) Localization of sutures at aortic wall (▲), adventitia (○), anulus (■), adventitia around avulsed commissure (★), coronary artery wall (●), respectively

Repair of aortic sinus: The previously trimmed normal aortic wall and a tailored fresh pericardial patch were sutured continuously, starting from the lowest point of the noncoronary sinus [Fig. 2A and B]. In order to treat the avulsion of aortic valve commissures, the annulus was sutured to the adventitia to resuspend and fasten the commissure, after which both were sutured to the pericardial patch [Fig. 2B]. Suturing was performed using the annulus itself instead of the intima near the annulus, as the former had a fibrous structure that provided more strength and reliability to the sutures.

**Fig. 3:**
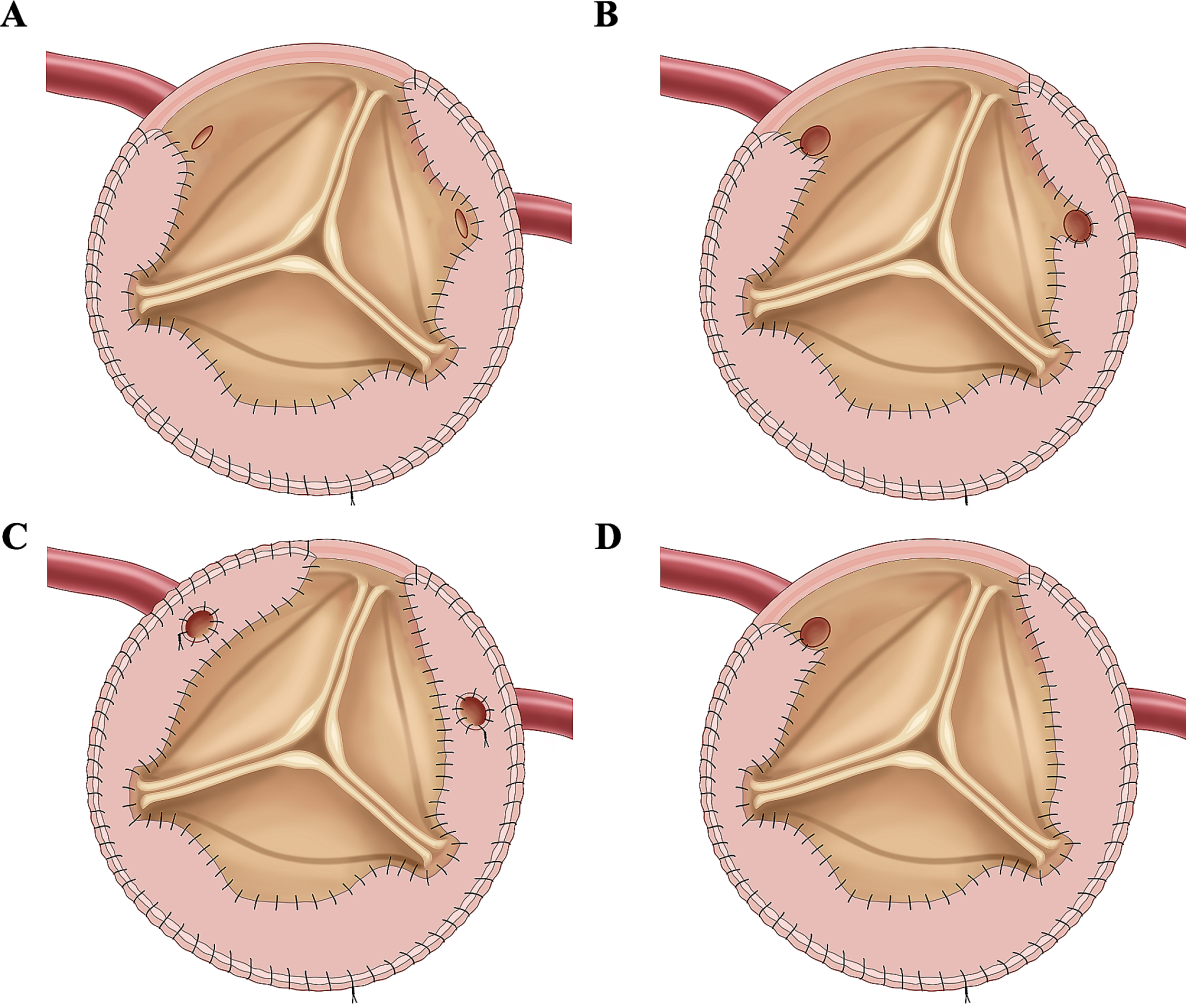
(**A**) to (**C**) Suture of aortic wall and pericardial patch where coronary ostia are not (**A**), partially (**B**) or totally (**C**) involved, and suture of annulus and adventitia at the avulsed commissures. (**D**) The closed coronary artery orifice is cut off from aortic lumen by the pericardial patch

Reconstruction of coronary ostia: The reconstruction method for coronary ostia primarily relied on the extent of aortic root dissection involving them. In cases where the coronary ostia remained intact, the dissected intimal flap was removed, and the normal aortic wall adjacent to the ostia was sutured to the pericardial patch [Fig. 3A]. However, if the coronary ostia were partially or completely affected by the dissection, the dissected intimal flap near the ostia was thoroughly excised, and the proximal normal coronary artery wall was sutured to the adventitia and then to the pericardial patch [Figs. 2B and 3B, and 3C]. Furthermore, if the coronary artery intima was transected or torn beyond the ostia, closure of the affected coronary artery orifice and a CABG were necessary [Fig. 3D].

Finally, the pericardial patch and adventitia were trimmed to the same height and continuously sutured together using a running 5 − 0 Prolene suture.

For patients in group B, we resuspended and fastened the commissures at the 3 commissural posts with 4 − 0 Prolene suture, and sutured the dissected two layers of the aortic root together at the sino-tubular junction using a running 5–0 Prolene suture circumferentially. The aortic sinus wall was reinforced around the coronary ostia in a “button” shape when the coronary sinus wall was dissected around the coronary ostia, while a CABG was performed when the dissection extended into the coronary arteries. The repaired aortic root was then anastomosed to the Dacron graft using a running 5 − 0 Prolene suture. Direct suture technique has been previously reported in detail by Yang and colleagues [6]. We performed direct suture in group B without using any biological glue or Teflon felts.

### Follow up

Patients were followed up by outpatient service or telephone for 1–60 months. Median follow- up duration was 24 months (Q1-Q3,10.75–44.25 months). Adverse events included all-cause death and reoperation for aortic disease. The follow-up rate is 100% and 96% in group A and group B, respectively.

### Study end points

The primary end points of the study were 30-day mortality and re-exploration for bleeding in overall patients.

### Statistical analysis

Continuous data were summarized as mean ± standard deviation for variables with a normal distribution, and as median and interquartile range for variables with a non-normal distribution. Categorical data were presented as frequencies and proportions. Comparisons of continuous variables were performed using Student’s t-test, while the Mann-Whitney U-test was used for non-normally distributed variables. For categorical data, the chi-square test or Fisher’s test was utilized. A multiple logistic regression was performed in order to adjust confounding factors and calculated odds ratio (OR) based on the following preoperative baseline characteristics: surgical technique, age, gender, preoperative serum creatinine (SCr), preoperative aortic valve insufficiency (AI), avulsion of aortic valve commissure, and disrupted coronary artery. Survival curves were generated using the Kaplan-Meier method, and survival rate comparisons between the two groups were performed using the log-rank test.

The stabilized inverse probability treatment weighting (IPTW) method was used to adjust baseline data. The propensity score (PS) was developed using multivariable logistic regression, while stabilized weights were calculated from the PS and used as weights for IPTW. The weight of patients was calculated using the formula Pt/PS for the group A and (1-Pt)/(1-PS) for the group B (Pt = the number of patients in group A/total patients). For comparing the group A and the B group, the PS model included age, gender, hypertension, coronary artery disease, preoperative SCr, preoperative AI, avulsion of aortic valve commissure, and disrupted coronary artery. From the divided differences in means by pooled SD and difference in proportions, we derived standardized mean difference between groups before and after IPTW matching. The absolute SMD was less than 0.2, representing the smallest possible difference between groups and a successful match. All statistical analyses were conducted using R software version 4.2.2, and statistical significance was defined as two-tailed *p*-values < 0.05.

## Results

### Patients characteristics and preoperative outcomes

A total of 95 patients who underwent aortic root repair were enrolled in the study, including aortic root repair using pericardial autograft (group A, *n* = 49) and direct suture (group B, *n* = 46). It was the same experienced surgeon that performed aortic root repair for both groups. The surgical period for group A spanned from 2019 to 2022, whereas group B underwent surgeries between 2017 and 2019. The mean patient age at the time of surgery was 54.4 ± 11.8 years, and 75% (*n* = 71) of the patients were males. There were no notable variations in the baseline characteristics between the two groups. Table 1 presents the baseline data, which encompassed age, sex, preoperative AI, preoperative aortic root diameter, and preoperative comorbidities such as hypertension, diabetes mellitus, and coronary artery disease. No significant differences were observed in these factors. Furthermore, parameters like SCr and alanine transaminase (ALT) did not exhibit any statistically significant differences (*P* = 0.430, *P* = 0.063, respectively).

**Table 1:** Demographic and preoperative data comparing the two groups

Items	Group A (*n* = 49)	Group B (*n* = 46)	*P*
Female	11(22)	13 (28)	0.678
Age (years)	53.7 ± 12.4	55.1 ± 11.2	0.568
BMI (kg/m^2^)	25.8 ± 4.2	26.6 ± 3.7	0.320
Hypertension	38 (78)	27(59)	0.926
Diabetes Mellitus	0 (0)	2(4)	0.232
CAD	7(14)	11(24)	0.350
Stroke	1(2)	3(7)	0.352
Previous cardiac surgery	1(2)	1(2)	1.000
Chronic kidney disease	1(2)	2(4)	0.609
Preoperative AI			0.159
None and trace	15(31)	18(39)	
Mild	20(41)	20(43)	
Moderate	6(12)	7(15)	
Moderately severe	2(4)	1(2)	
Severe	6(12)	0(0)	
Diameter of aortic root (mm)	38.5(37.0-41.5)	38.4(36.3–41.0)	0.723
Preoperative SCr (µmol/L)	96.0(76.0-114.7)	85.5(72.4-102.7)	0.430
Preoperative ALT (IU/L)	19.0(5.0–33.0)	23.0(15.3–37.0)	0.063

Values are mean ± standard deviation; n (%); or median (first quartile, third quartile). BMI: body mass index; CAD: coronary artery disease; AI: aortic valve insufficiency; SCr: serum creatinine; ALT: alanine transaminase

### Intra-operative outcomes and IPTW

The intra-operative data and postoperative data are described in Table 2. The rate of performing concomitant CABG in all patients was 34% (*n* = 32). In group A and group B, a total of 20 (41%) and 12 (26%) patients received a concomitant CABG, respectively. All patients had aortic arch involvement and the majority of patients in both groups underwent total arch replacement and frozen elephant trunk procedure. However, the cardiopulmonary bypass time for group A was longer than that of group B (222 vs. 173 min, *P* < 0.001). The same was the aortic cross-clamp time (170 vs. 115 min, *P* < 0.001). However, the duration of cardiopulmonary bypass and aortic cross-clamp in group A exhibited a tendency of declining annually, whereas in group B, no such discernible trend was observed (Figure S2). This phenomenon was probably because that aortic root repair using pericardial autograft was a new surgical technique, necessitating ample practice to enhance its execution speed.

**Table 2:** Intra-operative data and short-term outcomes

Items	Group A (*n* = 49)	Group B (*n* = 46)	*P*
Concomitant CABG	20(41)	12(26)	0.193
Aortic arch replacement			0.205
TAR + FET	44(90)	45(98)	
Partial arch replacement	5(10)	1(2)	
CPB time (min)	222.0(192.0-263.0)	173.0(152.0-200.0)	< 0.001
Aorta cross-clamp time (min)	170.0(145.0-195.0)	115.0(104.0-138.0)	< 0.001
Avulsion of aortic valve commissure			0.005
0	15(31)	12(26)	
1	15(31)	29(63)	
2	18(37)	5(11)	
3	1(2)	0(0)	
Disrupted coronary artery			0.001
0	12(24)	6(13)	
1	24(49)	38(83)	
2	13(27)	2(4)	
In-hospital mortality	0(0)	1(2)	0.484
30-day mortality	0(0)	3(6.5)	0.110
Re-exploration for bleeding	1(2)	3(7)	0.352
Renal failure requiring CRRT	5(10)	5(11)	1.000
New-onset stroke	3(6)	2(4)	1.000
Paraplegia	1(2)	1(2)	1.000
Postoperative AI			0.399
None and trace	35(76)	36(84)	
Mild	11(24)	7(16)	
Diameter of aortic root (mm)	36.3(34.0–38.0)	36.4(34.5–38.0)	0.895
Postoperative SCr (µmol/L)	154.2(111.8-289.1)	154.4(108.1-234.6)	0.594
Postoperative ALT (IU/L)	56.0(26.0-163.0)	57.0(42.3–147.0)	0.450

Values are mean ± standard deviation; n (%); or median (first quartile, third quartile). CABG: coronary artery bypass graft; TAR: total arch replacement; FET: froze elephant trunk;

CPB: cardiopulmonary bypass; CRRT: continuous renal replacement therapy; AI: aortic valve insufficiency; SCr: serum creatinine; ALT: alanine transaminase

Group A exhibited a higher number of aortic valve commissure avulsions (*P* = 0.005) and disrupted coronary arteries (*P* < 0.001) compared to group B. Therefore, the IPTW method was conducted in order to adjust baseline data. After IPTW, there were 49.95 patients in group A and 44.66 patients in group B with similar demographic and clinical characteristics and with reasonable absolute standardized mean differences, as shown in Table S1.

### Postoperative outcomes and short-term outcomes

The overall proportion of patients with coronary artery disrupted was 81% (*n* = 77). The study demonstrated an in-hospital mortality of 1% and a 30-day mortality rate of 3% among the patients included, and there was no significant difference in in-hospital mortality and 30-day mortality between the two groups (*P* = 0.484, *P* = 0.110, respectively). All patients who experienced premature death within 30 days were in group B. In group B, one patient with acute pericardial effusion died during hospitalization due to cardiogenic shock and multiple organ failure. The other two patients were discharged and died within 30 days, despite hemodynamic instability and the need for ventilator support. Among the two patients, one was diagnosed with mesenteric hypoperfusion syndrome, and the other with pericardiac tamponade and hypotension. There were no significant differences observed in postoperative adverse events, including re-exploration for bleeding (*P* = 0.352), new-onset stroke (*P* = 1), renal failure requiring continuous renal replacement therapy (CRRT) (*P* = 1), and paraplegia (*P* = 1), prior to discharge. Evaluation of postoperative AI and aortic root diameters using echocardiography also revealed no differences between the two groups (*P* = 0.399, *P* = 0.895, respectively). In both groups, the grade of AI was significantly improved (group A: *P* < 0.001; group B: *P* < 0.001) after surgery as shown in Figure S3 and Table S3. The aortic root diameters decreased significantly (group A: *P* < 0.001; group B: *P* = 0.005) after surgery as shown in Figure S4 and Table S4. Furthermore, there were no statistically significant differences in clinical laboratory parameters, such as postoperative SCr and ALT (*P* = 0.594, *P* = 0.450, respectively).

To further assess the postoperative outcomes, a multiple logistic regression analysis was conducted. The dependent variables were the primary endpoint events, encompassing 30-day mortality and re-exploration for bleeding. As shown in Table 3, the logistic regression analysis revealed a significant association between the primary endpoint events and surgical technique (odds ratio, 0.002; 95% confidence interval, 0–0.159; *P* = 0.026). Additionally, the postoperative outcomes after IPTW were shown in Table S2. The 30-day mortality in group A was lower than that observed in group B (*P* = 0.065), and the re-exploration for bleeding in group A was also lower than that in group B (*P* = 0.055). There were no significant differences observed in other postoperative adverse events, including new-onset stroke (*P* = 1), renal failure requiring continuous renal replacement therapy (CRRT) (*P* = 0.739). The primary endpoint events in group A (1.2%) were significantly lower than that observed in group B (12.5%) (*P* = 0.009).

**Table 3:** Logistic analysis regressions: the primary endpoint events

Items	Odds ratio	95%CI	*P*
Group A vs. Group B	0.002	(0.000,0.159)	0.026
Age per year	1.098	(0.941,1.377)	0.305
Female vs. Male	3.678	(0.200,106.838)	0.384
Preoperative SCr per µmol/L	1.027	(0.995,1.068)	0.108
The grade of preoperative AI	2.798	(0.748,19.605)	0.181
The number of aortic valve commissure avulsions	10.268	(0.473,600.579)	0.184
The number of disrupted coronary arteries	4.914	(0.256,300.088)	0.364

CI: confidence interval; SCr: serum creatinine; AI: aortic valve insufficiency

During the follow-up period of 30 days after surgery, there were three deaths in group A, while in group B one patient experienced premature death and two patients were lost to follow-up. There was no significant difference in short-term survival between the two groups after surgery (log-rank *P* = 0.75), as shown in Figure S5. Additionally, no patients in either group underwent reoperation for aortic disease or experienced more than moderate AI grade. There was no significant difference between the grade of AI (group A: *P* = 0.382; group B: *P* = 0.137) and aortic root diameter (group A: *P* = 0.119; group B: *P* = 0.962) at discharge and that of follow-up in both groups.

## Discussion

In this retrospective cohort study of patients underwent aortic root repair at our institution, we reported that the overall 30-day mortality was about 3%, while the early mortality of patients with ATAAD underwent emergent surgery was about 5-24% in most studies [8]. We speculate that the satisfactory perioperative outcome of this method is attributed to the relatively soft and elastic pericardial autograft, which may reduce the risk of perioperative proximal anastomotic bleeding and contribute to maintaining the physiological function of the aortic root. Another reason accounting for it is that the aortic root diameter of patients does not exceed 45 mm, which means the condition of their aortic root is relatively better than that of patients with aortic root diameter exceeding 45 mm.

The advantage of aortic root repair using pericardial autograft is the complete excision of the dissected intima, with only a remaining dissected intima confined to 5 mm from the avulsed aortic valve commissure. Additionally, we applied fresh pericardial autograft to reconstruct the aortic root without the using of any artificial materials or biological glue and then sutured it with the adventitia. We only resect the dissected intima but preserved the aortic root, which is different from valve-sparing aortic root replacement with either a reimplantation (David procedure) or remodeling (Yacoub procedure) technique, as described elsewhere in detail [9, 10]. The indications and contraindications of the two surgical techniques are largely different. In ATAAD patients with aortic root diameter exceeding 45 mm, it is necessary to replace aortic root with a composite biological or mechanical valve graft (Bentall procedure) or perform a David procedure or a Yacoub procedure [1]. On the contrary, we performed aortic root repair using pericardial autograft if the patient did not have an aortic root diameter > 45 mm or hereditary thoracic aortic disorders including Marfan syndrome.

Furthermore, we compared this new technique with the proven safe and effective direct suture technique for patients with ATAAD [6]. There was no significant difference in perioperative parameters. However, the extent of involvement of the aortic valve commissures and coronary ostia in group A was more critical than that of group B (*P* = 0.005, *P* = 0.001, respectively), indicating that the severity of aortic sinus damage was more pronounced in group A than in group B. The 30-day mortality was 0 and 6.5% for group A and group B, respectively. Although no significant differences were observed in 30-day mortality between the two groups (*P* = 0.110), the 30-day mortality in group A was nil. Our study showed that the severity of AI grade (group A: *P* < 0.001; group B: *P* < 0.001) and the enlargement of aortic root (group A: *P* < 0.001; group B: *P* = 0.005) were ameliorated in both groups after surgery [Figure S3 and S4], indicating both surgical techniques could improve the durability of aortic root and short-term effects, given AI and enlargement of proximal aorta could increase the risk of aortic root reoperation [11]. 

A multiple logistic regression analysis, considering these relevant confounding factors, indicated a significant correlation between the primary endpoint events and the surgical technique (OR, 0.002, 95%CI, 0–0.159; *P* = 0.026). This suggests that group A tended to have reduced 30-day mortality and a lower risk of re-exploration for bleeding, which was demonstrated by IPTW results. However, this improvement resulted in a longer operative time. The CPB time and aortic cross-clamp time in group A were longer than in group B. The extended duration of the operation suggests that aortic root repair using pericardial autograft may contribute to increased procedural complexity. Nonetheless, the clinical outcomes of our novel technique were satisfactory, without any increase in the incidence of adverse events. The results during the follow-up period showed that the aortic roots in both groups had good stability and durability, indicating that both surgical techniques have satisfactory short-term efficacy and could maintain the physiological function of the aortic root.

Our study explores the strategy of repairing coronary ostia when they are partially or totally involved by aortic dissection. Aortic dissection with coronary ostia involvement is a serious complication, the management of which lacks high-level evidenced-based medicine evidence [12]. At present, the mainly procedure to treat coronary ostia is closing the false lumen adjacent to them and then taking advantage of pericardial patch or Teflon felts to reinforce them in a “button” shape, or performing a CABG [1, 13, 14]. In our research, the overall proportion of patients with coronary ostia involvement was 81%. Besides, the rate of performing concomitant CABG in all patients was 34%, while that in previous reports ranged from 4 to 7.9% [6, 15, 16]. To explain this phenomenon, on the one hand, the coronary ostia of our patients might have been more severely involved by dissection because only patients with aortic root involvement were selected. On the other hand, we perform concomitant CABG when patients’ perioperative computer tomography angiography indicated severe coronary heart disease and heavy coronary artery calcification.

The choice of coronary artery treating strategy depended on the discretion of surgeons. Typically, if the coronary artery intima is transected or torn beyond the coronary ostia in both groups, the impaired coronary arterial orifice is closed and a coronary artery bypass graft is performed. Besides, in cases where the coronary ostia are involved, the strategies differ between group A and group B. In group A, the dissected intima flap adjacent to the coronary artery is removed until reaching the normal coronary ostia and the pericardial patch is used to reconstruct the coronary ostia, while in group B, surgeons mainly reinforce the aortic sinus wall around the coronary ostia using 5 − 0 Prolene as an in situ coronary button reimplantation as previously reported [6]. None in overall patients was diagnosed with perioperative acute myocardial infarction, the proportion of which is lower than that of previous reports [6, 17].

Although the clinical results demonstrate a satisfactory efficacy of the new technique, given the relatively small cohort and short follow-up period, this study has inevitable limitations. Firstly, we currently have not obtained long-term follow up data to demonstrate the long-term efficacy of this technique in patients who received aortic root repair using pericardial autograft. This study is ongoing and we will conduct further follow-up to collect long-term outcome data. Besides, our study has inherent bias due to its retrospective nature. In this study, we tended to perform our new surgical technique after 2020. The choice of surgical technique depended on the discretion of surgeons, making randomized study difficult. Additionally, although patients in group B underwent surgery from 2017 to 2019, we believed the operation and postoperative care of them were of a high standard, as evidenced by the consistency and comparability of the duration of cardiopulmonary and aortic cross-clamp with those reported in other studies [6]. Therefore, we thought that the impact of surgical year on postoperative and follow-up outcomes was minimal. Finally, because the follow-up of echocardiography was not 100% complete, we might underestimate the severity of follow-up AI grade and underestimate the length of follow-up aortic root diameter in both A and B groups.

## Conclusion

Both surgical techniques evaluated in our study were able to effectively repair aortic root, shorten the diameter of aortic root, and ameliorate AI. Besides, patients in group A tended to have reduced 30-day mortality and a lower risk of re-exploration for bleeding. Our findings suggest the safety and efficacy of aortic root repair using pericardial autograft for patients with ATAAD. This new surgical technique has satisfactory perioperative and short-term follow-up clinical outcomes and can serve as an alternative technique for facilitating aortic root reconstruction during emergent surgery of ATAAD.

### Electronic supplementary material

Below is the link to the electronic supplementary material.

Supplementary Material 1

## Data Availability

No datasets were generated or analysed during the current study.

## Abbreviations

ATAAD: Acute Stanford type A aortic dissection
CABG: coronary artery bypass graft
SCr: serum creatinine
AI: aortic valve insufficiency
ALT: alanine transaminase
CRRT: continuous renal replacement therapy
IPTW: inverse probability treatment weighting
OR: odds ratio

## References

[1] Malaisrie SC, Szeto WY, Halas M, Girardi LN, Coselli JS, Sundt TM 3 et al. rd,. 2021 The American Association for Thoracic Surgery expert consensus document: Surgical treatment of acute type A aortic dissection. J Thorac Cardiovasc Surg. 2021;162(3):735–758.e732. 10.1016/j.jtcvs.2021.04.053. 10.1016/j.jtcvs.2021.04.053 34112502

[2] Chang Y, Guo H, Qian X, Fang F (2020). A case report of aortic root repair using a pericardial autograft for type A aortic dissection. J Cardiothorac Surg.

[3] Tang Y, Liao Z, Han L, Tang H, Song Z, Xu Z (2017). Long-term results of modified sandwich repair of aortic root in 151 patients with acute type a aortic dissection. Interact Cardiovasc Thorac Surg.

[4] Tanaka K, Morioka K, Li W, Yamada N, Takamori A, Handa M (2005). Adventitial inversion technique without the aid of biologic glue or Teflon buttress for acute type a aortic dissection. Eur J Cardiothorac Surg.

[5] Rylski B, Bavaria JE, Milewski RK, Vallabhajosyula P, Moser W, Kremens E et al. Long-term results of neomedia sinus valsalva repair in 489 patients with type A aortic dissection. Ann Thorac Surg. 2014;98(2):582–588; discussion 588–589. 10.1016/j.athoracsur.2014.04.050. 10.1016/j.athoracsur.2014.04.050 24928674

[6] Yang B, Malik A, Waidley V, Kleeman KC, Wu X, Norton EL (2018). Short-term outcomes of a simple and effective approach to aortic root and arch repair in acute type a aortic dissection. J Thorac Cardiovasc Surg.

[7] Guo HW, Chang Y, Qian XY (2021). Aortic root repair using pericardial autograft for acute type a aortic dissection. J Thorac Cardiovasc Surg.

[8] Gudbjartsson T, Ahlsson A, Geirsson A, Gunn J, Hjortdal V, Jeppsson A (2020). Acute type a aortic dissection - a review. Scand Cardiovasc J.

[9] David TE, Feindel CM (1992). An aortic valve-sparing operation for patients with aortic incompetence and aneurysm of the ascending aorta. J Thorac Cardiovasc Surg.

[10] Yacoub MH, Gehle P, Chandrasekaran V, Birks EJ, Child A, Radley-Smith R (1998). Late results of a valve-preserving operation in patients with aneurysms of the ascending aorta and root. J Thorac Cardiovasc Surg.

[11] Ohno N, Maeda T, Kato O, Sato H, Ueno G, Yoshizawa K (2020). Neomedia Repair of the Valsalva Sinus in the treatment of Acute Type-A aortic dissection: long-term effectiveness and a case of Pathology. Ann Vasc Dis.

[12] Duan WX, Wang WG, Xia L, Xue C, Yu B, Ren K (2021). Clinical profiles and outcomes of acute type a aortic dissection and intramural hematoma in the current era: lessons from the first registry of aortic dissection in China. Chin Med J (Engl).

[13] Neri E, Toscano T, Papalia U, Frati G, Massetti M, Capannini G (2001). Proximal aortic dissection with coronary malperfusion: presentation, management, and outcome. J Thorac Cardiovasc Surg.

[14] Chen YF, Chien TM, Yu CP, Ho KJ, Wen H, Li WY (2013). Acute aortic dissection type A with acute coronary involvement: a novel classification. Int J Cardiol.

[15] Yang B, Norton EL, Hobbs R, Farhat L, Wu X, Hornsby WE (2019). Short- and long-term outcomes of aortic root repair and replacement in patients undergoing acute type a aortic dissection repair: twenty-year experience. J Thorac Cardiovasc Surg.

[16] Zhu C, Piao H, Wang Y, Wang T, Li D, Xu R (2019). A New Aortic Root reinforcement technique for Acute Type A aortic dissection surgery. Int Heart J.

[17] Hagan PG, Nienaber CA, Isselbacher EM, Bruckman D, Karavite DJ, Russman PL (2000). The International Registry of Acute Aortic dissection (IRAD): new insights into an old disease. JAMA.

