# Supplementary material for: Clinical outcomes of aortic root repair using pericardial autograft for acute type a aortic dissection
Source: J Cardiothorac Surg. 2024 Jun 26;19:379. doi: 10.1186/s13019-024-02909-2 (PMC11201898; doi:10.1186/s13019-024-02909-2)
Supplement: Supplementary file 1 — Supplementary Material 1 [file 13019_2024_2909_MOESM1_ESM.docx]

**Clinical outcomes of aortic root repair using pericardial autograft for acute type A aortic dissection**

**Supplemental Figure Legends**

**Supplemental Figure 1**


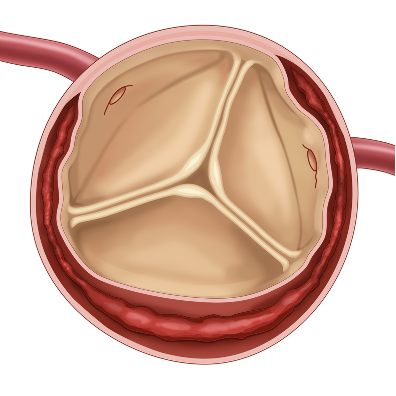


**Figure S1.** Aortic dissection with aortic root involvement can cause avulsion of aortic valve commissures and involvement of coronary ostia.

**Supplemental Figure 2**

**
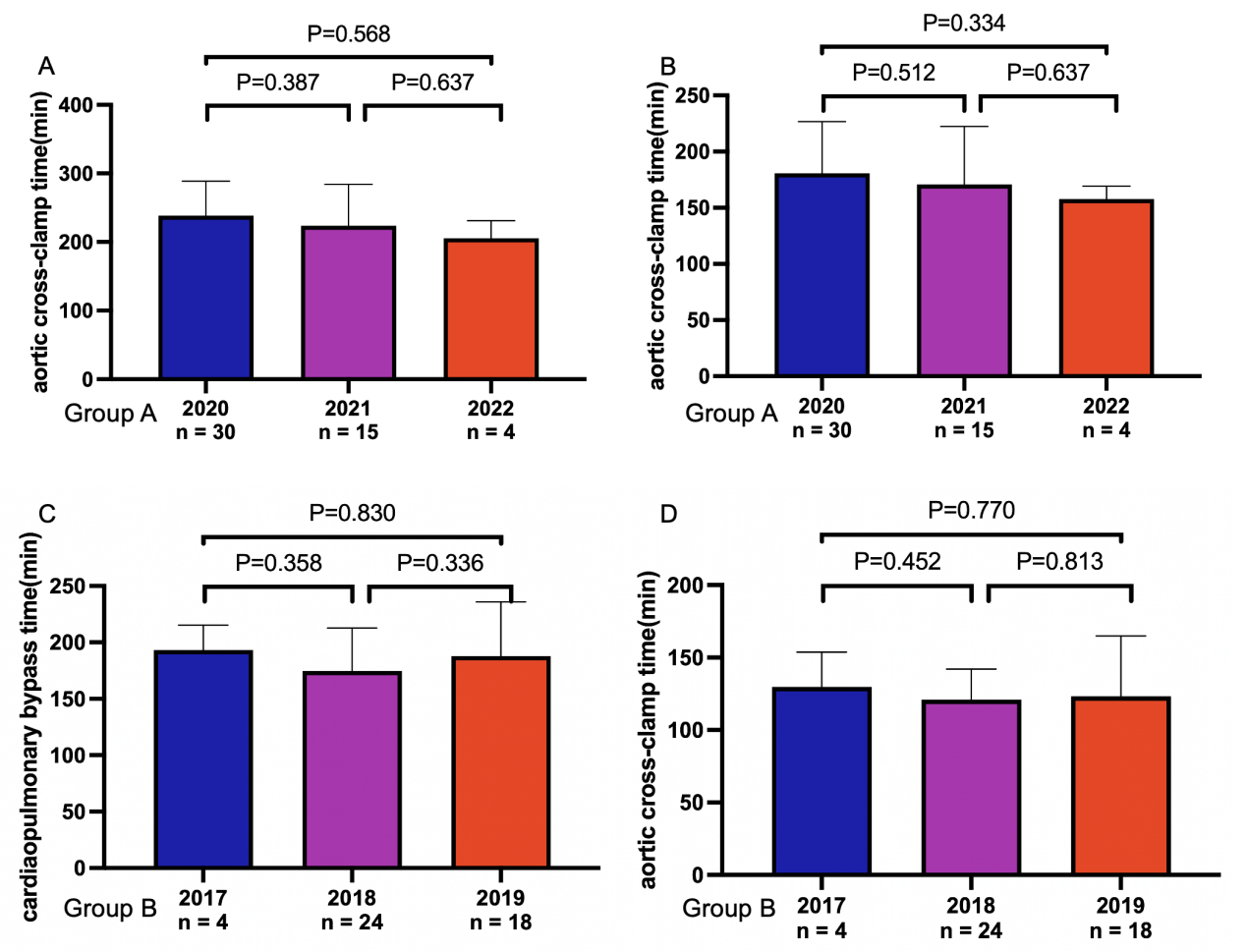
**

**Figure S2.** (A) to (D) The duration of cardiopulmonary bypass and aortic cross-clamp in group A (A and B) and in group B (C and D).

**Supplemental Figure 3**

**
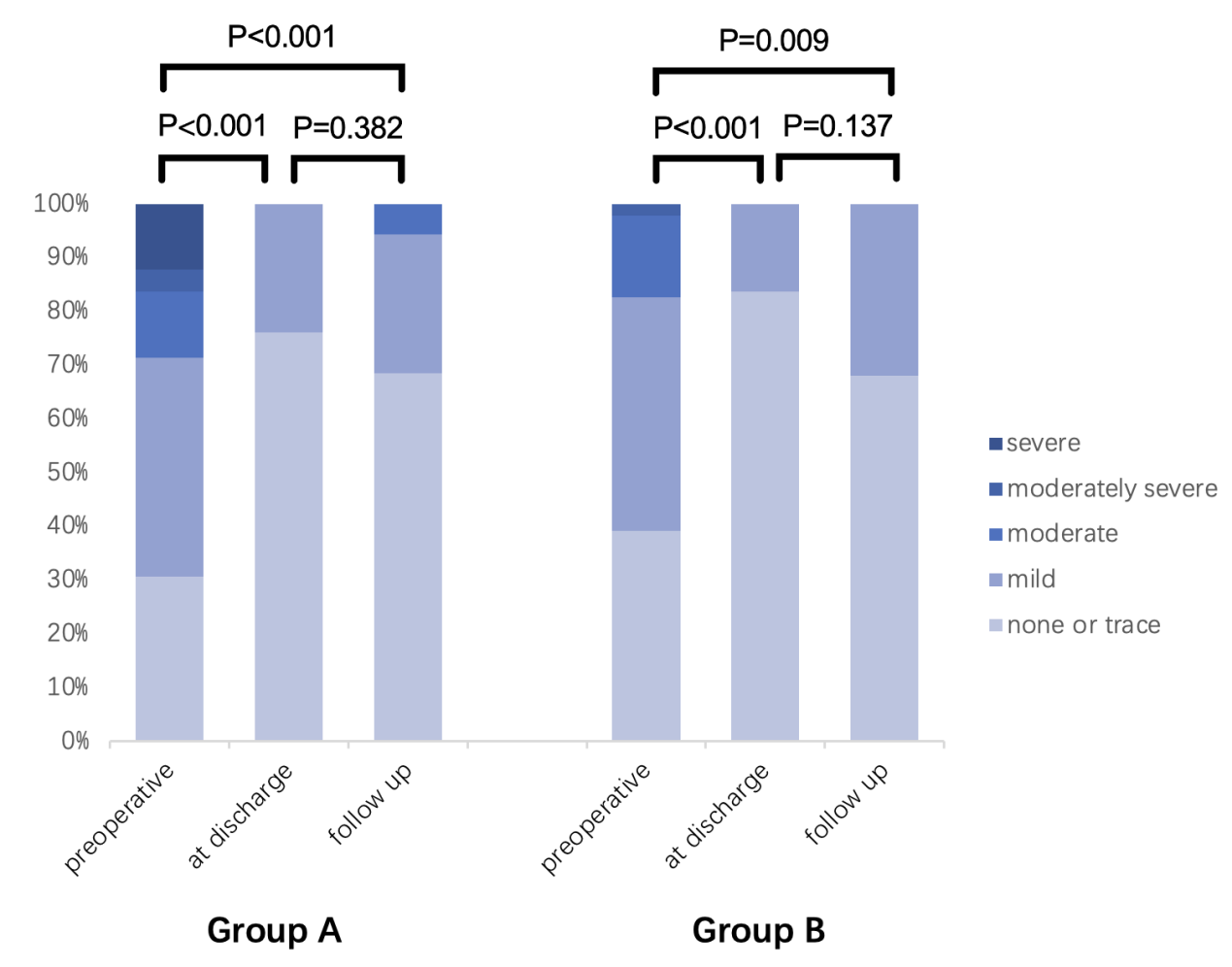
**

**Figure S3.** A comparison of aortic valve insufficiency before surgery, at discharge, and during follow-up between Group A and Group B.

**Supplemental Figure 4**

**Figure S4.** A comparison of aortic root diameter before surgery, at discharge, and during follow-up between Group A and Group B.

**Supplemental Figure 5**

**


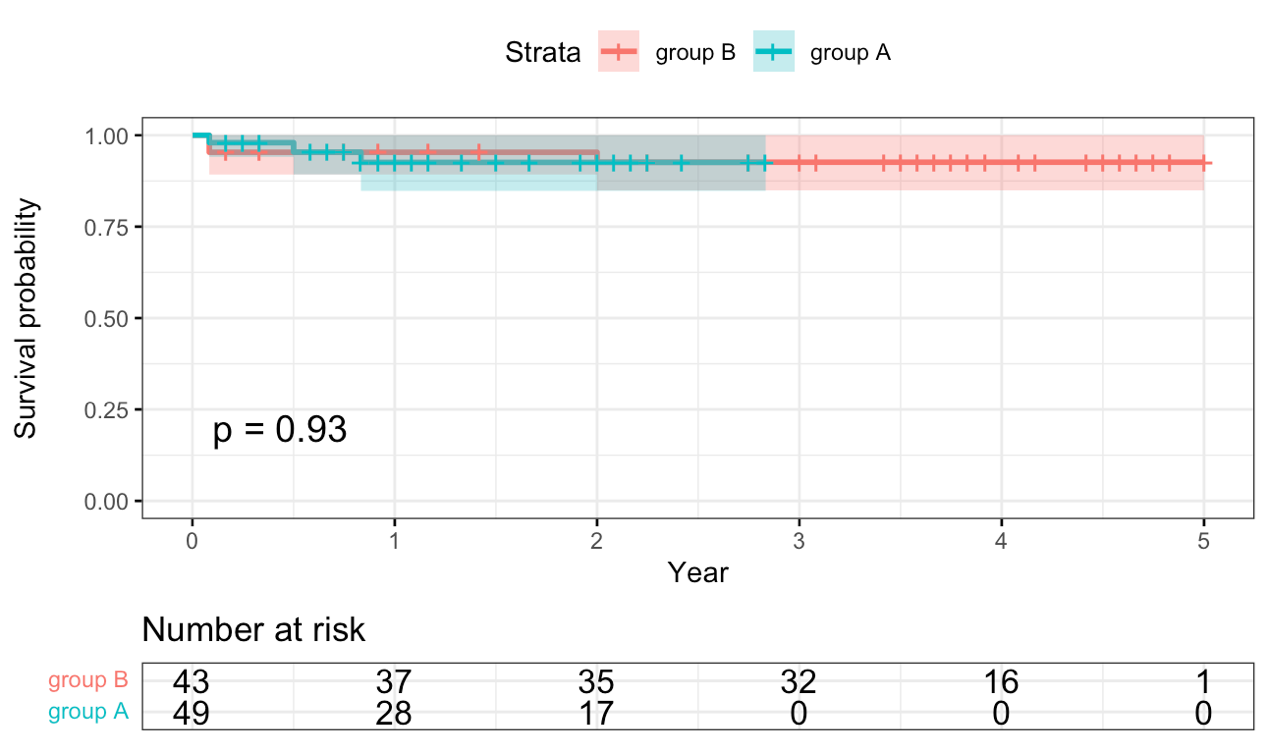
**

**Figure S5.** Kaplan-Meier survival analysis curves for two groups of patients (units: months)

**Supplemental Tables**

Table S1. Baseline characteristics of patients in group A and group B before and after IPTW.

|  | Before IPTW | | | |  | After IPTW | | | |
| --- | --- | --- | --- | --- | --- | --- | --- | --- | --- |
| Itemes | Group A | Group B | P | SMD |  | Group A | Group B | P | SMD |
| n | 49 | 46 |  |  |  | 49.95 | 44.66 |  |  |
| Age (year) | 53.7±12.4 | 55.1±11.2 | 0.568 | 0.118 |  | 54.8±12.8 | 54.6±11.0 | 0.950 | 0.014 |
| female | 11(22) | 13 (28) | 0.678 | 0.134 |  | 12.4(24.8) | 10.7(24.0) | 0.927 | 0.020 |
| hypertension | 38 (78) | 27(59) | 0.926 | 0.071 |  | 40.0 (80.0) | 35.7(80.0) | 0.999 | <0.001 |
| CAD | 7(14) | 11(24) | 0.350 | 0.247 |  | 11.4 (22.8) | 9.7 (21.8) | 0.913 | 0.025 |
| Preoperative SCr (μmol/L) | 96.0  (76.0-114.7) | 85.5  (72.4-102.7) | 0.430 | 0.259 |  | 89.9  (68.8-105.7) | 89.8  (72.5-110.8) | 0.863 | 0.033 |
| Preoperative AI |  |  | 0.159 | 0.554 |  |  |  | 0.290 | 0.480 |
| None/Trace | 15(31) | 18(39) |  |  |  | 19.1(38.2) | 14.8(33.2) |  |  |
| Mild | 20(41) | 20(43) |  |  |  | 20.6(41.1) | 19.6(44.0) |  |  |
| Moderate | 6(12) | 7(15) |  |  |  | 5.0 (10.1) | 8.6 (19.3) |  |  |
| Moderately severe | 2(4) | 1(2) |  |  |  | 1.5 (3.0) | 1.5 (3.5) |  |  |
| Severe | 6(12) | 0(0) |  |  |  | 3.8 (7.5) | 0(0) |  |  |
| The number of avulsions of aortic valve commissure | 1.10±0.87 | 0.85±0.60 | 0.102 | 0.341 |  | 0.92±0.87 | 0.91±0.60 | 0.919 | 0.022 |
| The number of disrupted coronary artery | 1.02±0.72 | 0.91±0.41 | 0.380 | 0.183 |  | 0.98±0.71 | 0.96±0.42 | 0.872 | 0.034 |

Values are mean ± standard deviation, n (%) or median (first quartile, third quartile). IPTW: inverse probability treatment weighting; SMD: standardized mean difference; CAD: coronary artery disease; SCr: serum creatinine; AI: aortic valve insufficiency.

**Table S2**. The postoperative outcomes after IPTW.

| **Items** | **Group A(n=49.95)** | **Group B(n=44.66)** | **P** |
| --- | --- | --- | --- |
| In-hospital mortality | 0(0) | 1(2.2) | 0.292 |
| 30-day mortality | 0(0) | 3.2(7.2) | 0.065 |
| Re-exploration for bleeding | 0.6(1.2) | 3.7(8.3) | 0.055 |
| Renal failure requiring CRRT | 4.4(8.8) | 4.8(10.8) | 0.739 |
| New-onset Stroke | 2.8(5.6) | 1.7(3.7) | 1.000 |
| Primary endpoint events | 0.6(1.2) | 0 | 0.009 |

Values are n (%) . CRRT: continuous renal replacement therapy; The primary endpoint events included the 30-day mortality and reoperation for hemostasis.

**Table S3.** The grade of aortic valve insufficiency

|  |  | Group A |  |  |  | Group B |  |
| --- | --- | --- | --- | --- | --- | --- | --- |
| Aortic valve  insufficiency | Preoperative  (n=49) | At discharge  (n=46) | Follow-up  (n=35) |  | Preoperative  (n=46) | At discharge  (n=43) | Follow-up  (n=25) |
| None or Trace | 15(31) | 35(76) | 24(69)  9(26) |  | 18(39) | 36(84) | 17(68) |
| Mild | 20(41) | 11(24) |  |  | 20(43) | 7(16) | 8(32) |
| Moderate | 6(12) | 0(0) | 2(6) |  | 7(15) | 0(0) | 0(0) |
| Moderately severe | 2(4) | 0(0) | 0(0) |  | 1(2) | 0(0) | 0(0) |
| severe | 6(12) | 0(0) | 0(0) |  | 0(0) | 0(0) | 0(0) |

**Table S4.** Aortic root diameter, median (first quartile, third quartile)

|  |  | Group A |  |  |  | Group B |  |
| --- | --- | --- | --- | --- | --- | --- | --- |
| item | Preoperative  (n=49) | At discharge  (n=44) | Follow-up  (n=29) |  | Preoperative  (n=46) | At discharge  (n=39) | Follow-up  (n=24) |
| Aortic root  diameter (mm) | 38.5  (37-41.5) | 36.25  (34-38) | 38  (35-41) |  | 38.35  (36.25-41) | 36.38  (34.5-38) | 37  (32.75-39) |
